# The time is now – a call to action for gender equality in global health leadership

**DOI:** 10.1017/gheg.2017.1

**Published:** 2017-05-24

**Authors:** R. Dhatt, K. Thompson, D. Lichtenstein, K. Ronsin, K. Wilkins

**Affiliations:** Women in Global Health, 30901 Wiegman Ct, Hayward, CA 94544, USA

**Keywords:** Gender equality, gender responsive, global health, Women in Global Health, women leadership in health, women's leadership, Women's Leadership in Global Health

## Abstract

Gender equality is considered paramount to the success of the Sustainable Development Goals and incorporated into global health programming and delivery, but there is great gender disparity within global health leadership and an absence of women at the highest levels of decision making. This perspective piece outlines the current gaps and challenges, highlighting the lack of data and unanswered questions regarding possible solutions, as well as the activity of Women in Global Health and efforts to directly address the inequity and lack of female leaders. We conclude with an agenda and tangible next steps of action for promoting women's leadership in health as a means to promote the global goals of achieving gender equality and catalyzing change.

## Introduction

Women comprise over 75% of the global health workforce in some countries and serve a crucial role in providing health to communities locally and globally [1]. Despite women holding the majority of lower to middle level positions, they are rarely seen at leadership and decision-making levels. This is particularly striking, as the global health community advocates for greater health equity and support of women's health, needs and rights. This commitment is enshrined in the Sustainable Development Goals (SDGs), which incorporate the attainment of gender equality, health and well-being for all people and the planet is in their contemporary vision of development including the target ‘Ensure women's full and effective participation and equal opportunities for leadership at all levels of decision-making in political, economic and public life’ [2]. To fully achieve these commitments leaders who espouse these ideals are essential.

Women in both politics and the business sector face similar problems. Gender inequality in leadership has proven an extremely difficult problem to solve and many strategies have been tried, but no consensus exists on how to move forward. Rwanda was the first country in the world to have more than 50% women in their parliament, and did this through the development of constitutional quotas [3]. Other strategies focus on pledges from world leaders and CEOs, such as the UN Women's HeForShe-movement [4], or the economic benefits to a corporation by having gender equality [5], to mention a few. The global health community champions further investment in girls and women, guided by evidence that that the needs of women and girls are often overlooked or inaccessible and that engagement of them leads to improved health for all [6, 7]. The global health community has worked to raise awareness and committed to addressing many of the challenges facing women, such as the gaps in the burden of disease, education, access to health services, nutrition and water/sanitation to name a few [8]. While advances have been made in these areas, significant gaps still exist. There is strong evidence that women's increased economic and political participation leads to a more developed and healthier world; however, the global health community has not done enough to ensure that the equality we strive to reflect in the planning and delivery of services is seen in the programmatic, policy and leadership levels [9]. Utilizing what we have learned from movements and groups in the past, and recognizing UN system initiatives such as the UN System Wide Action Plan for the Implementation of the CEB policy on Gender Equality and the Empowerment of Women and resolution WHA55.24 as points of progress, it is still believed that more needs to be done to lead to greater change, and more organizations must come together to address the gender leadership disparity in global health. Women in Global Health (WGH) is a movement with the goal of creating partnerships with such groups to work together to alleviate this leadership gender disparity.

## The scope of the problem

A review of leadership positions held by women in the top ten budgeted or independently ranked entities across seven sectors in 2016, specifically governments, intergovernmental agencies, foundations, private-sector, NGOs, global health initiatives and academia – showed massive disparities in gender parity [10]. This was particularly true when looking at political representation, academia and private-sector, three spheres which arguably have significant influence on society and health.

Gender disparities in leadership were also found in elected representation. In 2015, only 27% of Ministers of Health were women [11]. Similarly, only approximately 23% of chief delegates of Member State delegations at the 68th World Health Assembly were women. This disparity was relatively unchanged in 2016 at the 69th World Health Assembly, where only 26% of the chief delegates were women and moreover, evaluating the past 15 years the representation has averaged at 19% [11]. Meanwhile, the top ten grossing health-related companies in the private sector are led entirely by men. In academia, the seven of the deans at the top ten globally ranked schools of public health are men, and only 16% of medical school deans were women [10, 12]. This is despite the fact that women make up the majority of public and global health students. Harvard's global health student body, composed of 71% women, is a prime example of the gender disparity in academia [13]. It may be that this imbalance needs time; however, if we look at the largest medical student organization, International Federation of Medical Students' Associations only two out of the last ten presidents were women and only 15.6% historically presidents were women [14]

## Gaps and challenges

A great many questions regarding the role of women's leadership in global health remain. This avenue of inquiry is not well researched, analyzed and or understood within in global health. Some questions that still need to be investigated include: In a field dominated by women, why does the mid-level talent not rise through the pipeline to top leadership? Why are women in top leadership not visible? How should the global health community respond to the gender inequality in global health leadership? The global health community still needs the quantitative, disaggregated data and qualitative data that seeks to understand the gender dimension to confidently answer these questions, and to generate evidence-based policy and practice recommendations. In order to do this, the global health community must address current knowledge gaps on leadership and gender in global health, and be willing to commit to advancing gender equity within their organizations.

With the SDGs era upon us, it is imperative that the global health community recognize the importance of women at all levels and that there is a problem of inequality in our own community's leadership. It is time to start recognizing the Female Leadership Dividend, the evidenced understanding that societies with leaders reflecting their gender balance are greatly improved, [10] and harness all the potential in global health through striving for greater gender equity in global health.

## Women in Global Health

WGH is an independent movement working with partners at all levels to achieve greater gender equality in global health leadership. Our movement was conceived in Spring 2015, when we were asking ourselves Why do we still see all male panels in global health events and *what more can we do to elevate women*'*s contributions to global health and bring it center stage?*

In order to understand and address the disparity in leadership positions, WGH focuses on five strategic priority areas of: (1) raising awareness and understanding; (2) capacity building; (3) research and data collection; (4) policy analysis and recommendations; and (5) mentorship and networking. WGH works with other global health organizations and supporters to encourage stakeholders from governments, civil society, foundations, academia and professional associations and the private-sector to achieve gender equality in global health leadership and support gender mainstreaming. We work and coordinate with stakeholders and groups on fostering a culture of gender responsive leadership within their own organization, recognizing and understanding the lives, roles and contribution of women and men, ensuring that women benefit equitably from interventions and supporting its integration into their existing programs and capacities [15, 16]. We aim to elevate and support the role of women in this discipline, collaboratively with the involvement of men and all other genders through *raising awareness on the issue and creating gender responsive leaders in global health.*

WGH is currently partnered with initiatives such as Research in Gender and Ethics (RinGs), a research partnership, which seeks to galvanize gender and ethics analysis in health systems. Additionally, we have partnered with the Global Health Council, a membership organization connecting global health advocates and stakeholders, to develop the Women Leaders in Global Health Initiative (WLGHI). WLGHI is multi-year project focused on bringing awareness to the gender disparity in global health leadership, strengthen women's leadership roles and organizational capacity, and developing a broad network of people and organizations working together to create gender equality in leadership within the global health arena.

## A call to action

Together we call for the prioritization, development and advancement of gender responsive leadership to achieve gender equality in global health's top leadership by 2030. Our goal is to reach a minimum of 50–50 representation in women leadership in global health across all the key stakeholders and avenues. Having each global health leader make tangible public commitments and work toward achieving them, such as the measureable, achievable, realistic and results-based, time-bound (SMART) Commitments carried forward by the Geneva Gender Champions, a network of decision-makers working to breakdown gender barriers, will bring us ever closer toward achieving greater gender equity in global health.

Following a year of analysis and over 15 global dialogues, WGH has identified a comprehensive list of commitments that the global health community, particularly at the organizational and institutional level, need to commit to achieve the goal of greater gender equity in global health leadership. Further, WGH has begun to develop tools, as well as develop resources of well-established tools in partnership with key stakeholders, to better facilitate the translation of these commitments to action.

1. Leadership: Moving forward, the global health community should commit to leadership that is gender transformative and institutionalized. This is already being applied in other sectors through the leadership of global initiatives such as the UN Women and Planet5050.
2. Capacity building: All people, regardless of gender, working in the global health field and health sector and especially in leadership roles, should be required to go through a gender responsive training as part of a core competencies training.
3. Enabling environments: Create an environment which aims to recognize and increase the visibility of women's leadership in global health through – hosting gender balanced events, development of a recognition system and prioritization of active recruitment of women leaders as a goal in global health.
4. Mentorship and networking: It is imperative that mentorship be cultivated and supported early in training and profession, with greater investment in mentorship at the mid-career level when women leaders are at greatest risk of leaving the talent pipeline.
5. Research and data: Data should be disaggregated and reflexive in terms of sex and gender. Disaggregation of all health research, specifically accounting for sex and gender in the development of research questions, design experiments, analysis of data and reporting of results, particularly as it pertains to health systems governance.

We invite all of the broader global health community join us and we are calling on every stakeholder within the global health and development fields to take a critical look at their own organizations and to make commitments to advance women in global health leadership and achieve great gender equity in global health leadership at all levels.
